# Effect of the Post-Harvest Processing on Protein Modification in Green Coffee Beans by Phenolic Compounds

**DOI:** 10.3390/foods11020159

**Published:** 2022-01-08

**Authors:** Gustavo A. Figueroa Campos, Johannes G. K. T. Kruizenga, Sorel Tchewonpi Sagu, Steffen Schwarz, Thomas Homann, Andreas Taubert, Harshadrai M. Rawel

**Affiliations:** 1 Institute of Nutritional Science, University of Potsdam, Arthur-Scheunert-Allee 114-116, 14558 Nuthetal, Germany; figueroacamp@uni-potsdam.de (G.A.F.C.); johanneskruizenga@gmail.com (J.G.K.T.K.); sorelsagu@uni-potsdam.de (S.T.S.); homann@uni-potsdam.de (T.H.); 2 Coffee Consulate, Hans-Thoma-Strasse 20, 68163 Mannheim, Germany; schwarz@coffee-consulate.com; 3 Institute of Chemistry, University of Potsdam, Karl-Liebknecht-Str. 24-25, 14476 Potsdam, Germany; ataubert@uni-potsdam.de

**Keywords:** *Arabica* coffee, coffee processing, protein modification, bound phenolic compounds, peptide biomarkers, LC-MS/MS

## Abstract

The protein fraction, important for coffee cup quality, is modified during post-harvest treatment prior to roasting. Proteins may interact with phenolic compounds, which constitute the major metabolites of coffee, where the processing affects these interactions. This allows the hypothesis that the proteins are denatured and modified via enzymatic and/or redox activation steps. The present study was initiated to encompass changes in the protein fraction. The investigations were limited to major storage protein of green coffee beans. Fourteen *Coffea arabica* samples from various processing methods and countries were used. Different extraction protocols were compared to maintain the status quo of the protein modification. The extracts contained about 4–8 µg of chlorogenic acid derivatives per mg of extracted protein. High-resolution chromatography with multiple reaction monitoring was used to detect lysine modifications in the coffee protein. Marker peptides were allocated for the storage protein of the coffee beans. Among these, the modified peptides K.FFLANGPQQGGK.E and R.LGGK.T of the *α*-chain and R.ITTVNSQK.I and K.VFDDEVK.Q of *β*-chain were detected. Results showed a significant increase (*p* < 0.05) of modified peptides from wet processed green beans as compared to the dry ones. The present study contributes to a better understanding of the influence of the different processing methods on protein quality and its role in the scope of coffee cup quality and aroma.

## 1. Introduction

Coffee belongs to the global mainstream drinks [1,2]. The demand for coffee beverages is growing, and only crude oil has a larger trade volume [3]. Coffee processing is the process of isolating the green coffee bean by removing all remaining layers of the coffee cherry [4]. In general, coffee cherries are treated in two well established procedures: the dry and wet method. The more traditional method is dry processing. It is commonly practiced in sunny regions at lower altitudes [5] and is considered less technically demanding and less expensive. After harvesting, the ripe fruits are sun-dried, and depending on weather conditions, the drying process takes 12–15 days to reach the desired moisture content (10–12%) [6]. The dried husk composed of coffee pulp, mucilage, and parchment layer is removed subsequently by machine [6]. Coffee processed by the dry method is cherished for its fruity note and silky mouthfeel. Nevertheless, it is one of the most difficult methods to produce high-quality coffee [4]. In the past years, however, such natural processed coffees have been winning the highest prices in auctions (e.g., “Cup of Excellence”) and thus are considered meanwhile to have undergone high-quality processing. The wet method is performed in regions with humid climates and higher altitudes [7]. Machines and large quantities of clean water are used in the wet method. Removal of the coffee pulp layer is followed by fermentation [8]. Coffee fermentation takes between 24 and 36 h until the mucilage is degraded and separated from the bean [9]. Finally, the coffee beans surrounded by parchment and silver skin are dried in the sun or removed mechanically [10]. Wet-processed coffee has a full aroma with pleasantly higher acid content, and it is considered to be of higher quality compared to dry-processed coffee [4]. There are some hybrid forms of processing, namely the semi-wet and the semi-dry method, as well as monsooning. They differ in their method of mucilage removal. In the semi-washed method, the mucilage layer is removed after 1 day of sun-drying, whereas in the semi-dry, beans are placed to dry directly after removal of coffee pulp and are often referred to as “pulped natural” coffees [4]. In the case of the monsoon process, green coffee beans are stored in open warehouses for 6–7 weeks exposed to the warm and humid winds of the monsoon [11]. The “monsoon” approach should actually not be considered as a direct processing technique, but represents more of a post-processing method that is added after a natural dry process at the coast of Malabar.

The protein content of *C. arabica* green coffee beans lies between 8.5 to 12.7% [12]. Proteins are considered to be the most important fraction for the building of coffee aroma. During roasting, the Maillard reaction takes place [13], where free amino acids of proteins and peptides interact with carbonyl groups of reducing sugars. The final product consumed consists of 25% of the resulting melanoidins [12]. These account for the brown color and the aroma of coffee. Among the nitrogen compounds, free amino acids are particularly important for flavor development. The legume-like storage protein with sedimentation constant of 11S represents the main storage coffee bean protein fraction [14,15]. The 11S protein accounts for approximately 45% of the soluble protein fraction in the endosperm [14]. However, in a 2D electrophoresis, Rogers et al. [14] also found 11S protein in the embryo. The 11S storage protein generally contains a low content of sulfur. Thaler et al. [16] showed the content of methionine at 2.4% of amino acids in the 11S protein of the green Brazilian *C. arabica* beans. According to Rogers et al., only 1.1 and 0.2% of the amino acid content can be allocated to cysteine and methionine, respectively. Further, it was recently shown that the dry processing of the coffee beans may increase the content of the non-protein amino acid γ-aminobutyric acid [17]. In the soluble protein fraction of the 11S protein, glutamate and glutamine are the most abundant amino acids in coffee beans. Reducing SDS electrophoresis of the soluble protein fraction shows mainly three bands at 55, 33, and 24 kDa [12,15]. The 55 kDa belongs to the intact 11S globulin monomer, six of which combine together to form the hexameric structure of the native 11S storage protein. The monomer consists of *α*- and *β*-subunits. The two subunits are connected by a disulfide bridge. In an alkaline environment, the linked cysteine residues are susceptible to reduction, and separate bands appear for each of the 33 kDa and 24 kDa subunits [18,19].

The coffee beans are an excellent source of antioxidants, which include phenolic compounds such as chlorogenic acid [17,20], an ester formed between caffeic-and quinic acid. They appear in the form of caffeoylquinic acids (CQA), its corresponding three isomers (3-CQA, 4-CQA, and 5-CQA), dicaffeoylquinic acids (diCQA) with its respective three isomers (3,4-diCQA, 3,5-diCQA, and 4,5-diCQA), and feruloylquinic acids with the known three isomers (3-FQA, 4-FQA, and 5-FQA) representing further derivatives of hydroxycinnamic acids found in coffee beans [21]. Documented works report more than 50 hydroxycinnamic acid derivatives being present [22,23]. Depending on the species, green coffee contains 6–11% chlorogenic acid based on dry weight [10]. Proteins can be modified by reaction with phenolic compounds. The bulk of interactions between these two fractions initially result in non-covalent interactions [24]. These are based on hydrophobicity, hydrogen bonds, or ionic bonds [25]. Such modification can also occur in parallel with covalent reactions. This was shown by Prigent et al. [26] using the example of the reaction of 5-CQA with bovine serum albumin (BSA), lysozyme, and lactalbumin. Covalent bonds are catalyzed enzymatically and non-enzymatically. Both catalyzed reactions consume oxygen by oxidizing the phenolic compounds to quinone intermediates. Quinones are diketones that act as oxidants [27].Their double-bond leads to a positive partial charge inside the ring. In conjunction with nucleophilic groups of amino acid side chains, nucleophilic addition occurs [25,28]. In particular, the amine of lysine and the thiol group of cysteine are preferred reaction sites [29]. The reaction of a protein with a phenolic compound is mainly influenced by the nature of the two reaction partners [30]. Prigent et al. [26] showed that differences in hydrophobicity, isoelectric point, and amino acid sequence have a significant influence on the interaction of the protein. Regarding the phenolic compound, position and number of hydroxyl groups, molecular weight, and structural flexibility play an important role [27,30,31]. Reaction parameters such as exposure time, temperature, pH, and concentration of phenolic compounds also have an influence [27,32,33]. In addition, enzymes such as polyphenol oxidases, which catalyze the oxidation of phenolic compounds, drive the modification of proteins. In this context, the enzyme-assisted extraction from coffee beans was shown to have antioxidant, antityrosinase, and antimicrobial activities resulting from the polyphenol and peptide composition [34]. Protein extracts and bioactive peptides from green coffee beans and spent coffee grounds were also shown to have high anti-hypertensive and antioxidant potentials [35,36,37].

Based on the above evidence provided, the protein fraction is likely to be subjected to modifications even before coffee roasting. In the same context, the type of post-harvest processing may also play an important role in modulating the reaction of the amino acid residues with the phenolic compounds. The aim of this study is to contribute to the understanding of the influence of different coffee processing methods on such protein modifications. For that purpose, a high-resolution mass spectrometry method to detect modification of protein in the green coffee beans was developed. Samples of different processing methods and countries of origin were used to assess the modified peptides in the main storage protein of green coffee beans. 

## 2. Materials and Methods

### 2.1. Materials

Samples used in this work were of the species *Coffea arabica*. De-pulped coffee beans, initially fermented coffee beans, final fermented coffee beans, and green coffee beans were obtained from Rio Colorado Company (Palencia, Guatemala) and from Santa Sofia Company (Santa Rosa, Guatemala). In the Santa Sofia Company, part of the water is returned to the beginning of the process and used again in the de-pulping and fermentation steps. In the case of Rio Colorado Company, water is not recirculated, and freshwater is applied in the processing steps. The details of the processing conditions were described previously [9].

Green coffee beans from different processing post-harvest methods were also selected for this study, including the samples Maji (Ethiopia), Jora (Kenya), Mbeve Peak (Tanzania), Tarrazu (Costa Rica), Flaming (Mexico), Logoa and Santos (Brazil), Supremo (Colombia), Urubamba (Peru), Malabar (India), Kayumas, and Gayo (Indonesia). Semi-wet (Maji, Kayumas, Gayo); wet (Josra, Mbeye Peak, Tarrazu, Flamingo, Supremo, Urubamba); dry (Logoa, Santos); and Monsoon (Malabar) were purchased from Coffee well GmbH (Mettmann, Germany). These are commercially available samples, where the detailed data on post-harvest treatment were not further available. The analyzed samples were ground and fractionated to a particle size of <0.2 μm.

Proteomics-grade trypsin (Sigma Aldrich, Steinheim, Germany) or pepsin (Promega, WI, USA) were used to perform the in-solution digestion of the proteins. A synthesized peptide with the sequence GWGG (Bachem AG, Bubendorf, Switzerland) with a *m/z* ratio of 376.2 was used as an internal standard (IS) to normalize the data from the tandem mass spectrometry analysis. The in-silico digestion was performed by Skyline Software (MacCoss Lab Software, University of Washington; https://skyline.gs.washington.edu, accessed on 1 September 2020) [38]. The solvents used for mass spectrometry analysis were of LC-MS grade, and all the other chemicals were of analytical grade. 

### 2.2. Methods 

#### 2.2.1. Sample Preparation

For the composition of phenolic compounds, 10 mg of flours were mixed with 1 mL of 80% methanol in water (80:20, *v*/*v*). Extraction of polyphenols was performed at room temperature under shaking conditions for 30 min. After centrifugation at 9300× *g* for 10 min, supernatants were collected and stored at −20 °C.

Samples from two coffee plantations located in Guatemala were extracted by three different methods for protein determination. Extraction with polyvinyl polypyrrolidone (PVPP; option I) was based on Laing et al. [39] and Ali et al. [18]. The extraction protocol with sodium dodecyl sulfate (SDS; option II) was developed as described by Want et al. [40] and Figueroa et al. [9]. Extraction protocol option II was improved and used for further analysis. A schematic flowchart of the three extraction protocols is provided in Figure S1 (see Supplementary Materials).

Briefly, for method I: 100 mg of powdered sample and 50 mg of PVPP were mixed with 1 mL of 0.04% ascorbic acid in water (*v*/*v*). Extraction was performed at room temperature under shaking conditions for 2 h. After centrifugation at 4000× *g* for 20 min, supernatants were collected and stored at −20 °C. For method II: 100 mg of powdered sample was mixed with 1 mL of n-hexane under shaking conditions for 10 min. After centrifugation at 7000× *g* for 10 min, supernatants were discharged. Subsequently, 1 mL of n-hexane was added and the lipids re-extracted and discharged again, and samples were then allowed to dry in opened microtubes for 10 min at room temperature. Finally, 750 µL of SDS buffer were added to each sample and heated for 20 min at 50 °C. After centrifugation at 7000× *g* at 4 °C for 5 min, supernatants were collected. Method III consisted of mixing 20 mg of powder sample with 1 mL of n-hexane under shaking conditions for 10 min. After centrifugation at 7000× *g* at 4 °C for 10 min, supernatants were discharged. Thereafter, the precipitates were washed again with 1 mL of n-hexane and left to dry in opened microtubes for 1 h at room temperature. Subsequently, 750 µL of SDS buffer (50 mM Tris-HCl with 2% SDS at pH 6.8) were added to each precipitate. Then 20 µL of 0.25 M of tris-(2-carboxyethyl) phosphine (TCEP) solution were briefly mixed with the precipitates and next heated at 50 °C for 20 min in the dark. Subsequently, 20 µL of 0.25 M of iodoacetamide (IAA) solution were added to the precipitates and incubated at 50 °C for another 20 min in the dark. Mixtures were afterward centrifuged for 5 min. Finally, the supernatant was transferred to a new microtube, and precipitates were discharged. 

Protein fractions extracted with the three methods were finally mixed with 1 mL of acetone at 4 °C and incubated at −20 °C for 20 min. After centrifugation at 7000× *g* for 5 min, supernatants were discharged. Right after, 1.5 mL of methanol at 4 °C was added and mixed for 20 s and incubated for 20 min at −20 °C. After centrifugation at 7000× *g* for 5 min, supernatants were transferred to new microtubes.

After extraction with methods I and II, samples were mixed with 500 µL of 4 M urea buffer containing 0.1 M ammonium bicarbonate under shaking for 1 min followed by 10 min of ultrasonic treatment. Mixtures were centrifuged at 10,000× *g* for 5 min, and supernatants were stored at −20 °C.

#### 2.2.2. Free Amino Nitrogen

Free amino nitrogen was performed to measure the concentration of free amino groups according to the previously described procedure [41]. The samples were extracted using method III and subsequently dissolved in 4 M urea solution. Glycine stock solution (2 mg/L) was used as standard. Forty microliters of the mixed solution or standard was diluted in distilled water. Briefly, 400 µL of the diluted solution was mixed with 200 µL of ninhydrin staining reagent (containing 1 g Na_2_HPO_4_, 0.6 g KH_2_PO_4_, 50 mg Ninhydrin, and 30 mg Fructose in 10 mL distilled water). The resulting ninhydrin mixtures were heated at 100 °C for 16 min, solutions were cooled down to 20 °C for 20 min and 1 mL of 12 mM potassium iodide solution was added. The absorbance against distilled water was measured using the corresponding spectrophotometer (Jenway Genova, Staffordshire, UK). Absorbance was measured at 570 nm. The content of free amino nitrogen was calculated using equation 1 and the results expressed as µg/mg protein:

FAN = (A_S_ − A_B_ − A_C_)/(A_G_ − A_B_) × 2 × F (1)

where, A_S_ is the absorbance of the sample, A_G_ is the absorbance of glycine standard solution, A_B_ is the absorbance of the blank, A_C_ is the absorbance of the corrected blank, F is the dilution factor, and 2 is the concentration of glycine standard solution (mg/L).

#### 2.2.3. Free Thiol Groups

Free thiol groups were measured to examine the modification of cysteine side chains that is induced by the oxidation reaction of phenolic compounds. Reduced glutathione and N-acetylcysteine were used for the calibration curves. For the determination of free thiol groups, the extraction was performed as per method III, but without reduction and alkylation steps. The extracted pellet was dissolved in Tris buffer (0.2 M) after acetone precipitation and methanol purification. For the determination of all total free thiol groups, on the other hand, the pellet was dissolved in 0.2 M Tris buffer containing 1% SDS. Briefly, 450 µL of the dissolved samples or standards were mixed with 30 µL of (5,5′-dithiobis-(2-nitrobenzoic acid) (DTNB) in the cuvettes while using the spectrophotometer (Jenway Genova, Staffordshire, UK). The mixtures were incubated at room temperature for 10 min and absorbance was measured at 421 nm. The results are expressed as nmol SH groups/mg protein.

#### 2.2.4. Determination of the Composition of Protein-Bound Phenolic Compounds 

The composition of the phenolic compounds was determined using an HPLC system (Shimadzu HPLC system GmbH, Leonberg, Germany) [9]. Analyzes were performed with a C8 column (250 × 3.0 mm, particle size 5 µm, at 37 °C; MZ-Analysetechnik GmbH, Mainz, Germany) with a pore size of 300 Å. Undigested samples extracted by the improved SDS extraction method (Option III, Figure S1, Supplementary Material) were used. After extraction, the protein pellets were dissolved in 0.5 mL urea extraction buffer and separated at a flow rate of 0.6 mL/min for 20 min. The eluents were 0.1% trifluoroacetic acid in distilled water (eluent A) and acetonitrile (eluent B). The gradient was as described in the following: 0% eluent B from 0.01 to 3 min; 40% eluent B, from 3 to 7 min; 40% eluent B, from 7 to 10 min; 80% eluent B from 10 to 11 min; 80% eluent B, from 11 to 13 min; and 0% eluent B from 14 to 20 min. The detection was monitored at 280/325 nm for the determination of phenolic compounds (with a UV-Vis SPD-10 AVP detector, Shimadzu, Kyoto, Japan). The results were expressed as µg phenolic compounds/mg protein. 

#### 2.2.5. Determination of Protein Content 

The different extracted samples were compared for their protein content by using the method of Lowry et al. [42] with Bovine Serum Albumin (BSA) as standard. 

#### 2.2.6. Fluorescence Spectroscopy

Fluorescence spectroscopy was performed to evaluate browning reactions occurring during the extraction. Samples were dissolved in 0.5 mL 0.4 M urea extraction buffer. Samples were diluted at 1:1280 with the same extraction buffer. The light emitted in the range of 300–500 nm light wavelength was recorded with a Spectro fluorophotometer (Shimadzu, Duisburg, Germany) using an excitation wavelength of 280 nm. The signal intensity was determined by using the area under the curve for the emitted light (AUC). Pure urea extraction buffer was used for the blanks and subtracted from the AUC of the samples.

#### 2.2.7. Sodium Dodecyl Sulfate-Polyacrylamide Gel Electrophoresis 

Sodium Dodecyl Sulfate-Polyacrylamide Gel Electrophoresis (SDS-PAGE) was performed to determine the resulting molecular weight change as described previously [9]. Briefly, samples were first reduced using Novex™ NuPAGE™ LDS sample buffer (ratio of sample/buffer; 1:1, *v*/*v*) and then heated at 95 °C for 10 min. Ten microliters of the solution were then introduced in the wells of a Novex™ NuPAGE™ 4–12% Bis-Tris gels (Thermo Scientific™, Carlsbad, CA, USA). Spectra Multicolor Broad Range Protein-Markers (Thermo Fisher Scientific, Vilnius, Lithuania) were used for molecular weight calibration. The separation was performed for about 2 h at 30 mA. After staining overnight in Coomassie Blue G250 solution, the gels were destained using distilled water containing 10% acetic acid for 2 h. Finally, the gels were scanned (Bio-5000 Professional VIS Gel Scanner, SERVA Electrophoresis GmbH, Heidelberg, Germany) and analyzed with ImageLab software (Bio-Rad Laboratories GmbH, Feldkirchen, Germany). 

#### 2.2.8. In-Gel Digestion 

SDS-PAGE was performed as described above with the proteins stained with a colloidal Coomassie brilliant blue G 250 solution [43]. Selected gel bands belonging to the 11S coffee storage proteins obtained from the SDS-PAGE were cut with a scalpel and placed in a 0.5 mL microtube. Thereafter, 200 µL of destaining solution (80 mg ammonium bicarbonate in 40 mL acetonitrile/water mixture–50%/50%–*v*/*v*) was added and incubated at 37 °C for 30 min. Subsequently, 100 µL of tris-(2-carboxyethyl)-phosphine (TCEP) reduction buffer (25 mM) was added and incubated at 60 °C for 10 min. Next, 100 µL of iodoacetamide (IAA) buffer (25 mM) was mixed and incubated at room temperature for 1 h in the dark. IAA buffer was removed, and 200 µL of the destaining solution was added and incubated at 37 °C for 15 min. One-hundred microliters of acetonitrile were added and incubated at room temperature for another 15 min. Acetonitrile was removed, and gel pieces were allowed to dry for 15 min. The digestion was performed by using trypsin and pepsin enzymes. Digestion using trypsin: The treated gel pieces were mixed with 15 µL of activated trypsin. After incubation at room temperature for 15 min, 25 µL of 25 mM ammonium bicarbonate buffer was added. Digestion using pepsin: The treated gel pieces were mixed 15 µL of activated pepsin. After incubation at room temperature for 15 min, 25 µL of 0.09 M HCl was added. Finally, gel pieces were macerated manually and incubated in the dark for 20 h.

#### 2.2.9. MALDI-TOF-MS

Samples digested for 20 h were placed in an ultrasonic bath for 10 s (BANDELIN electronic GmbH, Berlin, Germany) and subsequently centrifuged for 10 min. After centrifugation, the supernatant was removed and transferred to a fresh reaction vessel. The matrix solution was freshly prepared by mixing 20 mg of *α*-Cyano-4-hydroxycinnamic acid with 300 µL of acetonitrile and 700 µL of 0.1% TFA. Two microliters of the samples were mixed with 2 µL of matrix solution, and 3 µL of the matrix-sample mixture was placed on a MALDI plate for each sample. Peptide calibration standard II (Bruker, MA, USA) was used as standard. The matrix-sample mixtures were crystallized at room temperature after 20 min. The time-of-flight analyzer (Autoflex Speed, Bruker, MA, USA) was run with the software FlexControl 3.4 (Bruker). After calibration, 30–40% of laser intensity was applied to the crystallized matrix samples until clear peaks were recognized. The mass spectra obtained were analyzed using the software FlexAnalysis 3.3 (Bruker). The background noise was minimized by using the baseline subtraction function. Calibration was performed by using the spectra of the closest standard to the sample position. For each spectrum, a mass list of the peaks with high intensity was exported to the software BioTool 3.2 (Bruker). From there, the detected peptide masses were compared with the Swiss Prot database using the Mascot interface. The parameters of the Mascot database search were set with the selected enzyme (pepsin or trypsin) at 0–2 partials, modification carbamidomethyl, and mass tolerance at 500 ppm. In addition, the FASTA sequence format of the 11S storage protein (P93079_COFAR, UniProt database) was used in the Sequence Editor of the BioTools software to simulate the in silico digestion with trypsin or pepsin, respectively [18]. FASTA is a program for searching and comparing protein and/or DNA sequences [44]. The fragment pattern obtained was in turn compared with the mass lists of the MALDI-TOF-MS spectra.

#### 2.2.10. In-Solution Digestion 

For the tryptic digestion, the extracts after acetone precipitation were re-dissolved in 400 µL of digestion buffer containing 0.1 M ammonium bicarbonate. After the addition of 20 µL of the trypsin solution (4 mg/mL), incubation at 37 °C under shaking conditions for 20 h was performed. After the addition of 15 µL of 40% formic acid (to denature the enzyme and stop the reaction), samples were cleaned using the solid phase extraction process before performing the LC-MS/MS analysis.

#### 2.2.11. Solid-Phase Extraction 

Solid-phase extraction (SPE) was performed to remove any impurities of the extracted samples. For that purpose, C18 material (300 mg, Chromabond C18 ec, Marchery-Nagel, Düren, Germany) was placed into a glass column. After activating the column (with 6 mL of 50% acetonitrile), the columns were then equilibrated with distilled water containing 0.1% formic acid. Digested samples were then applied onto the column and then washed with 6 mL of distilled water. The analytes were finally recovered using 1 mL of acetonitrile solution containing 0.1% of formic acid, filled to 5 mL using distilled water, and transferred into the vials for the LC-MS/MS analysis.

#### 2.2.12. Development of a Multiple Reaction Monitoring (MRM) Assay Using HPLC-MS/MS

**Figure 1:**
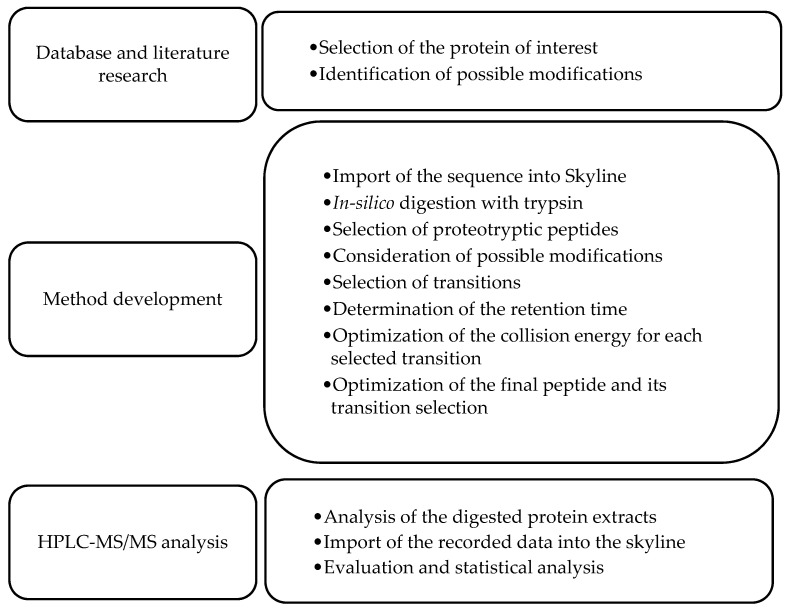
Method development for the relative quantification of the peptides by LC-MS/MS.

For the analysis of protein modification in connection with phenolic compounds, an MRM method was developed using HPLC-MS/MS. The method focuses on the detection of unmodified and lysine-modified peptides of the *α*- and *β*-chain of the trypsin-digested 11S protein present in protein extracts of *C. arabica*. For the method development, a defined workflow was followed, and the corresponding steps are shown in Figure 1.

The information of the 11S protein sequence was downloaded from the website of UniProt database (https://www.uniprot.org, accessed on 2 September 2020). A total of four unreviewed records with information obtained from literature and curator-evaluated computational analysis was compiled from the UniProt database. The comparison/alignment of the sequences is given (Supplementary Figure S2). Based on previous work, the choice among the listed options was made for P93079_COFAR [18].

The selected sequence in FASTA data file format was opened in the MacCross Lab Skyline Software. An in-silico digestion was performed with a list of all possible peptides, considering the probability of their formation, while referring to their ionization capability, signal strength, and specificity [45]. The following settings were applied: Trypsin was selected as the proteolytic enzyme. The maximum number of missed cleavage sites (partials) was set to zero. No background proteome was set. Peptides with 5–25 amino acid residues were selected for the analysis, while the carbamidomethyl modification of the cysteine residues by iodoacetamide was considered. The transitions were filtered for double-charged precursors and single-charged fragment ions. In addition, only fragmented y-ions were monitored. For each precursor peptide, transitions of up to six fragment ions were analyzed. While using preliminary collision energy for the individual transitions, the intensity was determined. Thereafter, the collision energy was optimized. For this purpose, different collision energies were tested for each intact mass and the one that achieved the largest peak area after the MS/MS run was selected. The selected transitions of the *α* and *β*-chain are listed (Supplementary Table S1). To measure lysine-modified masses, a structural modification of an additional mass of 352 m/z for the CQA monomer and a mass gain of 682 m/z for the DiCQA (dimer) was allocated for thiol and lysine groups in Skyline (Supplementary Figure S3) [18]. The modification by DiCQA is relevant only for the interaction with the amino side chains [18].

An HPLC-MS/MS Agilent 1260 system (Agilent Technologies Sales & Services GmbH & Co. KG, Waldbronn, Germany) equipped with an Agilent G6470A series triple Quad LC/MS (Agilent Technologies Sales & Services GmbH & Co. KG, Waldbronn, Germany), integrated with an electrospray (ESI) source operating in positive and negative ionization mode, was used to perform the analysis of the tryptic digested protein from coffee beans. one microliter of each analyte solution was separated through a Kinetex C8 analytical column (150 × 4.60 mm, 2.6 µm, 100 A; Phenomenex, Torrance, CA, USA) using a flow rate of 0.5 mL/min. The column was thermostated at 30 °C. Water containing 0.1% formic acid and 100% acetonitrile were used as eluent A and eluent B, respectively, under the following conditions: 100% solvent A from 0 to 5 min, 50–5% solvent A from 20 to 24 min, and 100% solvent A from 25 to 28 min. The desolvation gas temperature in the ionization source was set to 275 °C, gas flow rate of 11 L/min, nebulizer pressure of 35 PSI, fragmentor voltage of 130 V, and dwell time of 20 s. Nitrogen was applied as collision gas. Multiple reaction monitoring (MRM) mode was selected as the method for the detection, where a specific transition was monitored at a specific retention time. The separation time that ranged from 3 to 20 min was set for the MS-data collection, and the relative abundances of the targeted compounds were determined by using the total area of all the transitions. 

#### 2.2.13. Molecular Modeling Experiments

The general methodology for the modeling of the coffee 11S storage protein, template searching, 3D model building, validation, and model refinement was performed as described by Ali et al. [18]. The sequence of P93079_COFAR 11S storage globulin (UniProt online database; https://www.uniprot.org, accessed on 1 Decenber 2021) was used for homology modeling, and the accessibility of two main reaction sites was simulated by molecular modeling to illustrate the eligibility of the detected CQA modifications.

#### 2.2.14. Data Analysis

All experiments were performed in triplicate, and data are expressed as mean ± standard deviation. The results were analyzed with GraphPad Prism 8^®^ (GraphPad Software, Inc., San Diego, CA, USA) while applying two-way ANOVA and Tukey’s test, using a statistically significance set at *p* < 0.05.

## 3. Results and Discussions 

### 3.1. Free Thiol Groups and Amino Nitrogen in the Protein Fraction 

Free thiol groups and free protein-bound amino groups were monitored to determine their reactivity and change during processing. These were determined for coffee beans processed from two pilot wet-processing companies from Guatemala, and results are shown in Table 1. Exposed free thiol groups from coffee beans processed in Santa Sofia samples are found in high quantities. The values of 19.32 ± 1.23, 19.56 ± 1.25, 16.13 ± 1.03, 13.47 ± 0.86, and 5.91 ± 0.38 nmol/mg protein were obtained for de-pulped coffee beans, initial and final fermentation, washed, and green coffee beans, respectively. Conversely, with Rio Colorado samples, values of 8.00 ± 0.51, 14.43 ± 0.92, 2.61 ± 0.17, 13.74 ± 0.88, and 4.93 ± 0.31 nmol/mg protein were obtained. The amount of free thiol groups in the presence of SDS reports significantly different values (*p* < 0.05) for each group in Santa Sofia and Rio Colorado samples. The presence of SDS denatures the proteins, but also improves its solubility. In that case of denaturation and unfolding of the protein, an increase of the thiol values occurs, whereby a higher solubility leads to lower thiol levels. This results in slight fluctuations as documented in Table 1. The values of 7.65 ± 0.13 and 4.57 ± 0.076 nmol/mg protein were obtained for green coffee beans in Santa Sofia and Rio Colorado Company, respectively. These results allow the assumption that cysteine residues are less affected by the post-harvest treatment of coffee beans from Rio Colorado Company, where water is not recirculated, and freshwater is applied in the processing steps. In the Santa Sofia Company, part of the water is returned to the beginning of the process and used again in the de-pulping and fermentation steps, eventually allowing an increase due to microbial load or a more pregnant denaturation of the coffee proteins via enzymatic and/or redox activation steps, which would allow a release of free thiol groups. The identification of the underlying mechanisms represents an element of future studies.

The values of the measurements of free amino nitrogen (FAN) ranged from 38.15 ± 1.14 and 48.62 ± 3.86 µg/mg protein, which were obtained for the green coffee and final fermentation coffee beans processed in Rio Colorado Company. The content of FAN for green coffee beans processed in the Santa Sofia sample decreased significantly (*p* = 0.148) compared to de-pulped coffee beans, and values were 35.93 ± 4.14 and 46.97 ± 1.17 µg/mg protein for green and de-pulped coffee beans, respectively. High free amino nitrogen values indicate that samples analyzed have more unmodified lysine side chains that could be available for the initial Maillard reaction during roasting.

### 3.2. Protein Content

To compare the different extracted samples, protein content was determined according to Lowry et al. [42]. Table 2 shows the results of the protein content determined after extraction with PVPP (option I), SDS (option II), and the improved SDS extraction protocol (option III).

It is clearly observed that extraction using option II achieves significantly higher protein content for Rio Colorado Company samples compared to extraction using option I (*p* < 0.0001). Protein content was found to be 1.33 ± 0.21 and 2.08 ± 0.08 mg/100 mg DW for Rio Colorado samples extracted with option I and II, respectively. Values of 1.62 ± 0.04, and 1.73 ± 0.19 mg/100 g DW were obtained for Santa Sofia samples extracted with option I and II, respectively. Samples extracted with option III exhibited the highest content in protein, 3.75 ± 0.04 and 3.45 ± 0.13 mg/100 g DW for the Rio Colorado and Santa Sofia Company, respectively. The results indicate that protein content using SDS in the extraction protocol was higher compared to those using PVPP and ascorbic acid.

A fluorimeter was used to measure the visible fluorescence spectrum of the protein extracted with the three different option protocols. The dissolved extracts, especially after extraction with option I, show a darker green color compared to those of option II and III. Protein extract with option III was colorless compared to option I and II (Supplementary Figure S4). Bongartz et al. [46] reported a change of color when sunflower protein in an alkaline environment was incubated with CQA. An adduct e.g., with lysine results in a green benzacridine derivative as reported in [47,48] and is confirmed with the aid of HPLC coupled with ESI-MS^n^ [49]. If the mechanism proposed by Namiki et al. is followed [47,48], a dimerization prior to the interaction with proteins seems to be a precedent as documented for chlorogenic acid [26], and validated for the adduct formation with the amino group in a model system [25,49]. Thereafter, the present data suggests that the extraction with PVPP induces oxidation of phenolic compounds during the extraction and gives unexpected results by the added protein modifications. The extraction with SDS showed less influence on the analyzed protein fraction; therefore, option III was selected as a method for further analysis. Transmission measurements using a spectrophotometer from 325 to 480 nm wavelength were performed to quantify the different color appearances. The analysis of the variance showed that differences were significant (*p* < 0.05) for the two types of process. Option III, either for Santa Sofia or Rio Colorado processing, showed the lowest emission values as well as the highest protein content; therefore, it was chosen for further experiments. A clue to the loss of coloration could also lie in the structural changes occurring in the lysine adducts of CQA.

**Figure 2:**
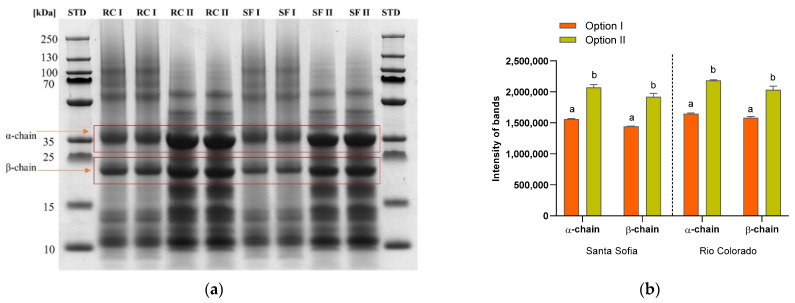
(**a**) SDS-PAGE of Rio Colorado and Santa Sofia Company green coffee beans; (**b**) intensity of *α* and *β* chains. RC: Rio Colorado Company; SF: Santa Sofia Company. Data are expressed as means± standard deviation (*n* = 3). Different letters indicate significantly different values for each group (*p* < 0.05, ANOVA, Tukey’s test).

SDS-PAGE was used to compare the extraction protocols (the option I and II), and the results are presented in Figure 2a. The main coffee 11S storage protein was allocated to *α*- and *β*-chain bands as indicated in Figure 2a. The band intensity after extraction with option II was significantly higher (*p <* 0.05) compared to the extraction with option I, indicating a higher efficiency of the protein extraction (Figure 2b). The 11S storage protein is composed of three to six monomers of masses of 150−400 kDa, which migrate into storage vacuoles and create, by hydrophobic interactions, the tri- and hexameric quaternary forms [50]. The removal of the disulfide bonds under reducing conditions in the 11S protein monomers releases the *α* (acidic) and *β* (basic) subunits [19]. *Coffea arabica* proteins, in non-reduced state, showed subunits with 55 kDa, and in the reduced state (2-mercaptoethanol) two sub-fractions with 33 and 24 kDa [14,15]. The presented data confirm the role of 11S storage protein as the most abundant one in green coffee.

### 3.3. In-Gel Digestion 

The gel bands of the *α*- and *β*-chain of the samples were cut, and digestion either with trypsin or pepsin was performed. The sequence coverage of peptic and tryptic in-gel digestion is given (Supplementary Figure S5). Fragment spectrum obtained by MALDI-TOF-MS analysis was compared to the sequence of the in-silico digested 11S. The fragment spectrum of the pepsin digestion with zero partials covers 3.6 and 4.3% for *α*-chain and *β*-chain, respectively. The sequence coverage increased significantly (*p* < 0.05) if a further partial was allowed in the digestion. Values of 19.5 ± 1.6 and 22.8 ± 1.6% were obtained for *α*-chain and *β*-chain, respectively. If two partials were allowed, the sequence coverage increased significantly to 35.3 ± 11.0 and 33.2 ± 12.1%. The sequence coverage of the MALDI-TOF-MS fragment spectra of the trypsin-digested samples does not change significantly with an increase in the partials. Figure S5b (Supplementary Material) shows the sequence coverage of the samples after extraction with options I and II. There is no significant difference between the two extraction options. The sequence coverage of the *α*-chain shows lower values compared to those of *β*-chain for Santa Sofia and Rio Colorado. Samples were measured only one time, therefore, differences cannot be statistically checked for significance. These preliminary experiments indicate that tryptic digestion is more effective and was therefore used for further experiments.

### 3.4. Phenolic Substances in Protein Extract by HPLC

**Figure 3:**
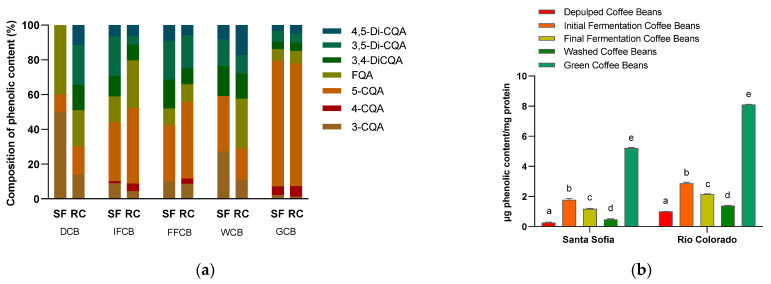
Phenolic compounds in protein extract. (**a**) Composition of the major phenolic compounds expressed as percentage of total phenolic compounds; (**b**) amount of phenolic compounds shown as the sum of means ± standard deviation of the detected phenolic compounds (*n* = 3). SF: Santa Sofia Company; RC: Rio Colorado Company; DCB: de-pulped coffee beans; IFCB: initial fermentation coffee beans; FFCB: final fermentation coffee beans; WCB: washed coffee beans; GCB: green coffee beans. Different letters indicate significantly different values for each group (*p* < 0.05, ANOVA, Tukey’s test).

Phenolic compounds in protein extracts were determined by HPLC to investigate the content of the phenolic substances as influenced by the post-harvest treatment. The percentage distribution of the seven detected phenolic compounds (Figure 3a) differs from the two coffee companies, especially between de-pulped coffee bean samples. The structures of these seven main compounds are given in our former work [18]. Recent studies determined more than 50 hydroxycinnamic acid derivatives [22,23]. The complexity of following up this type of reaction in coffee-based food matrix arises from the fact that the major phenolic compounds present in coffee beans are liable to isomerization and oxidation, thus producing themselves a series of reaction products, many of which have hardly been characterized [25]. The constituents 3,4-DiCQA, 3,5-DiCQA and 4,5-DiCQA were not detected in de-pulped coffee beans for Santa Sofia Company. Otherwise, the similar composition was detected for the initial fermentation, final fermentation, and washed and green coffee beans. The predominant constituent was 5-CQA with 72.3 and 70.7% for green coffee beans from Santa Sofia and Rio Colorado Company, respectively. The protein extraction (Section 2.2.1) indicates that the proteins were precipitated by the addition of acetone and thereafter washed with methanol. This treatment can remove the loosely bound phenolic compounds, but only the subsequent treatment with urea (desolvation of the protein) and separation under conditions of the chromatographic conditions allow the release of those molecules that are more tightly bound to the proteins, as indicated in Figure 3. It can be inferred that these phenolic compounds detected in the protein extract entered in non-covalent interactions with the proteins. The composition of phenolic compounds of green coffee beans shows similar composition to coffee beans processed in Santa Sofia or Rio Colorado Company. Individual constituents were added up to obtain an approximation of the total phenol content and data are shown in Figure 3b. The highest content of phenolic compounds was found in the green coffee beans with concentrations of 5.21 ± 0.04 and 8.11 ± 0.02 µg/mg protein for Santa Sofia and Rio Colorado Coffee Company, respectively. Thus, the drying step during the post-harvest treatments promotes a stronger binding of the phenolic compounds. The results further indicate that coffee bean processing by Rio Colorado Company triggers stronger binding of the proteins compared to Santa Sofia Company. Again, the post-harvest treatment seems to play a significant role, allowing the hypothesis that the proteins may undergo a more thorough denaturation via enzymatic and/or redox activation steps due to the re-use of water in Santa Sofia Company processing. This in turn could be responsible for structural changes in the storage protein, resulting in a loss of binding sites, thereby allowing a lower binding of the phenolic compounds.

### 3.5. Analysis of the Protein Modification Using HPLC-MS/MS

Proteins can be modified by reaction with phenolic compounds. The bulk of interactions between these two fractions initially result in non-covalent interactions [24,51]. These can be based on hydrophobicity, hydrogen bonds, or ionic bonds [25]. Suryaprakash et al. (2000) showed that proteins from sunflower seeds can act as non-covalent ligands for caffeic and quinic acids [52]. In this context, these interactions often occur between caffeic acid and tryptophan, tyrosine, or lysine side chains [52]. If the interaction of protein and phenolic compound leads to a covalent bond, it is a protein modification. Such a modification can also occur in parallel with non-covalent reactions. Covalent bonds are catalyzed both enzymatically and non-enzymatically. Both reactions can be divided into two steps and require the presence of oxygen [25]. Generally, the first step involves the formation of an electrophilic reactive species of *o*-quinone. These are capable of undergoing a nucleophilic addition to proteins e.g., thiol and free amino groups, thereby covalently modifying the proteins. The green coffee beans have been shown to possess polyphenol oxidase (PPO) activity [17]. Therefore, their proteins are liable to this type of modification [18]. In order to access such modifications, the proteins need to be broken down into peptides. Thus, a method was developed as indicated in Section 2.2.11 to encompass such changes. The strategy includes a first step of identifying the unmodified peptides in the major 11S coffee storage protein and a second one that follows a modification by a single or dimerized chlorogenic acid molecule while applying targeted mass spectrometric analysis [18].

**Figure 4:**
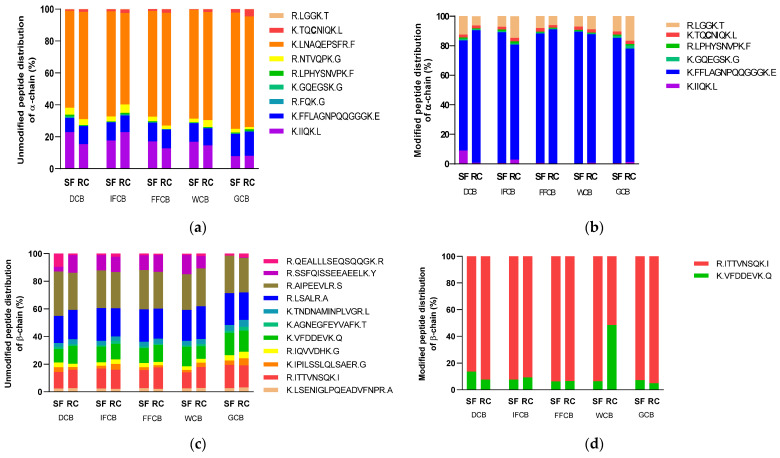
Distribution of unmodified and modified peptides of the *α*-chain (**a**,**b**) and *β*-chain (**c**,**d**) detected by HPLC-MS/MS from coffee samples from Santa Sofia (SF) and Rio Colorado (RC) Coffee Company. DCB: de-pulped coffee beans; IFCB: initial fermentation coffee beans; FFCB: final fermentation coffee beans; WCB: washed coffee beans; GCB: green coffee beans.

The percentage distribution of the detected masses for the *α* and *β*-chain of 11S protein can be seen in Figure 4. The peak areas of the fragment masses were added for each transition. It can be seen that the fragment K.LNAQEPSFR.F gave the strongest signal. The distribution values were between 57.3 and 72.8% for Rio Colorado initial fermentation sample and Santa Sofia green coffee beans, respectively. The modified peptides are dominated by the peptide K.FFLAGNPQQQGGGK.E. This peptide was found modified with the CQA monomer. The corresponding proportion of the modified peptides of the *α*-chain ranges from 74.5% to 90.7% for Santa Sofia de-pulped coffee beans and Rio Colorado final fermentation, respectively. The peptide R.LGGK.T was also detected in the modified state. This peptide was found modified with CQA and DiCQA. 

The distribution of unmodified peptides of the *β*-chain is depicted in Figure 4c. More homogenous distribution is observed compared to the distribution in the *α*-chain. In fact, the response for the *β*-chain was much stronger and more peptides could be allocated, in turn improving the overall sequence coverage.

From Figure 4d, it can be seen that only two modified peptides were detected. The peptide R.ITTVNSQK.I with DiCQA lysine modification is the predominant peptide in almost all the samples. Washed coffee beans in Santa Sofia show a proportion of 48.5% K.VFDDEVK.Q with a CQA-lysine adduct, differing significantly from the rest of the samples investigated. The reason for this observation is not yet clear and further experiments are needed to confirm this behavior.

**Figure 5:**
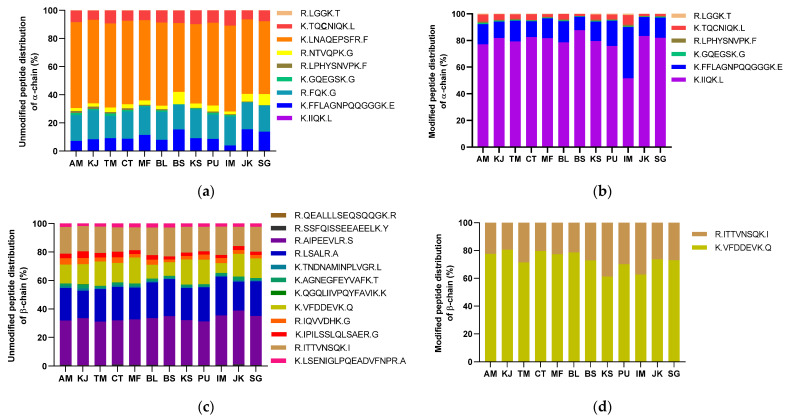
Distribution of the unmodified and modified peptides of the *α*-chain (**a**,**b**) and *β*-chain (**c**,**d**) of 11S by HPLC-MS/MS. AM: Maji Ethiopia; KJ: Jora Kenia; TM: Mbeye Peak Tanzania; CT: Tarrazu Costa Rica; MF: Flaming Mexico; BL: Logoa Brazil; BS: Santos Brazil; KS: Supremo Colombia; PU: Urubamba Peru; IM: Malabar India; JK: Kayumas Indonesia; SG: Gayo Indonesia.

The HPLC-MS/MS method was also applied to protein extracts from green *C. arabica* beans of various origins. The distributions of the unmodified and modified peptides of the *α*-chain are depicted in Figure 5a,b. Among the modified peptides, K.IIQK.L was found to be predominant. The lysine-modified R.LGGK.T peptide was found in all the samples.

In the case of the proportion of modified peptides in the *β*-chain (Figure 5d), Brazil Santos green coffee beans (BS) are particularly marked by the peptide R.ITTVNSQK.I with 20.2%. In the case of Mexico Flamingo (MF), the unmodified peptide K.VFDDEVK.Q shows a percentage distribution of 18.3%. The most modified peptide of the *β*-chain in all samples was K.VFDDEVK.Q. The proportions of the modified peptides of the *β*-chain are lower compared to that of the *α*-chain.

**Figure 6:**
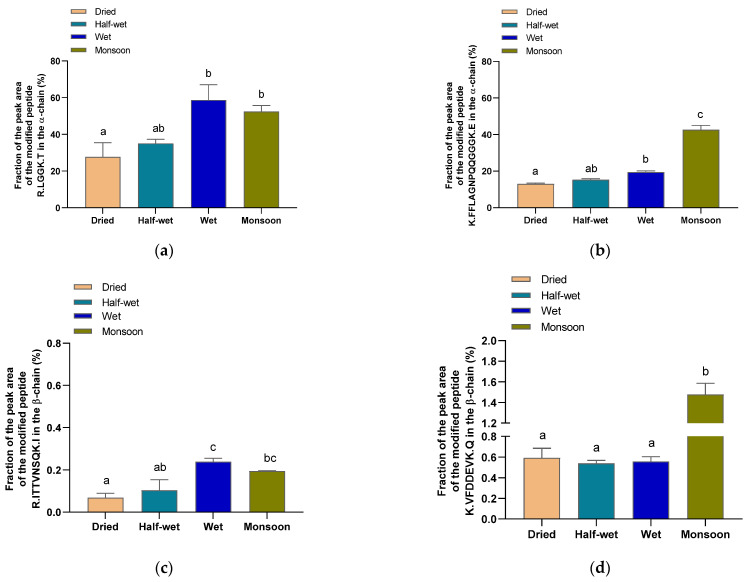
The proportion of modification of lysine residues in selected peptides of the *α*-chain (**a**,**b**) and *β*-chain (**c**,**d**) of the 11S storage protein as determined by HPLC-MS/MS. Different letters indicate significantly different values for each group (*p* < 0.05, ANOVA, Tukey’s test).

The proportions of modified peptides in the *α*-chain are shown in Figure 6a,b as mean values for each type of coffee processing. The percentage of modified peptide R.LGGK.T in green coffee beans has a higher trend for the wet and the monsoon compared to the dried and the half-wet processing. The situation was similar with the modified peptide K.FFLAGNPQQGGK.E in green coffee beans. A significant increase in the peak area of the modified peptide fraction was observed among the dried (13.0%), wet (19.5%), and monsoon processing (42.6%). In the case of *β*-chain (Figure 6c,d), it can be seen that the proportion of modified R.ITTVNSQK.I peptide is higher in the wet compared to the half-wet processing. In the case of the peptide K.VFDDEVK.Q, a significantly different (*p* < 0.05) proportion is seen in monsoon compared to the dried, half-wet, and wet processing.

**Figure 7:**
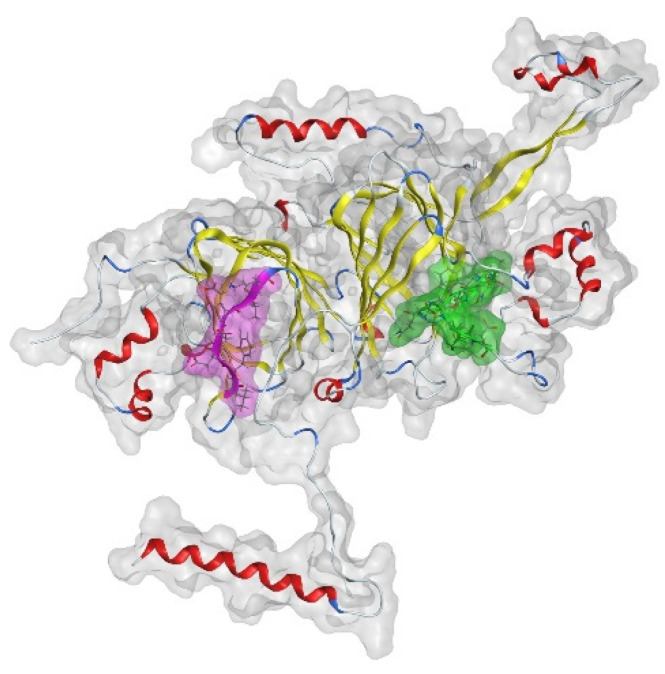
Visualization of the position of selected modified peptides K.FFLAGNPQQQGGGK.E (*α*-chain; green-colored segment) and K.VFDDEVK.Q (*β*-chain, pink-colored segment) in the monomer of coffee 11S storage protein.

The peptides K.FFLANGPQQGGK.E and R.LGGK.T of the *α*-chain show a high level of lysine modifications in the samples analyzed. In the case of the *β*-chain, peptides were less lysine-modified. Schwenke et al. [53] and Rawel et al. [32] showed that the hydrophilic C-terminal region of the *α*-chain on the surface of the 11S contained a protective function against the internal *β*-chain, thereby being the preferred point of attack for chlorogenic acid. The modeling of selected modification sites for the two chains of the coffee 11S protein monomer is given in Figure 7. The results of these simulations indicate the accessibility of the two reaction sites K.FFLAGNPQQQGGGK.E on the *α*-chain and K.VFDDEVK.Q on the *β*-chain, which are determined to be two of the most modified peptides out of those detected. Interestingly, the modification does not hinder the digestion by trypsin. Green coffee samples produced by different processing methods showed a connection with CQA-dependent lysine modification. Further work should confirm the amino acid involved in these reactions. Samples of the half-wet, wet, and monsoon processing contain larger proportions of the modified target peptides. Presumably, the increased contact with water favors the oxidation reactions under consideration.

## 4. Conclusions

This study was initiated to encompass the changes taking place in the protein fraction of coffee beans during different post-harvest processing steps. Three different extraction protocols to isolate the protein fraction were compared. Subsequently, a method to detect lysine and cysteine modification in coffee protein was developed. The unmodified peptide K.LNAQEOSFR.F of the *α*-chain was detected with high signal intensity. The unmodified peptides of the *β*-chain showed a diverse spectrum and, therefore, several options for selecting peptides as markers were available. The peptides K.FFLANGPQQGGK.E and R.LGGK.T of the *α*-chain showed a high level of lysine modifications in the samples analyzed. In the case of the *β*-chain, peptides were less modified. Recirculation of water during coffee processing led to more lysine modification. The main output of these preliminary experiments indicates that post-harvest treatment does affect the protein quality, and a larger sample set needs to be analyzed to validate the observed trends and changes. Further work will be directed to the possibility of using the corresponding peptides with more in-depth analysis of further modifications, in order to establish them as biomarkers while also bringing them closer in relation to coffee cup quality. 

## Figures and Tables

**Table 1:** Exposed free thiol, total free thiol groups, and free amino nitrogen in coffee beans from Santa Sofia and Rio Colorado Company.

		DCB	IFCB	FFCB	WCB	GCB
Santa Sofia	Exposed free thiol groups (nmol/mg)	19.33 ± 0.78 ^a^	19.56 ± 0.12 ^a^	16.13 ± 0.66 ^b^	13.47 ± 0.42 ^b^	5.91 ± 2.62 ^c^
	Total free thiol groups (nmol/mg)	23.20 ± 0.24 ^a^	14.52 ± 1.48 ^b^	15.90 ± 0.21 ^c^	10.13 ± 1.20 ^d^	7.65 ± 1.20 ^e^
	Free amino nitrogen (µg/mg)	46.97 ± 1.18 ^a^	48.51 ± 3.80 ^a^	57.52 ± 4.87 ^b^	58.96 ± 6.86 ^c^	35.94 ± 4.15 ^d^
Rio Colorado	Exposed free thiol groups (nmol/mg)	8.00 ± 0.45 ^a^	14.44 ± 0.48 ^b^	2.61 ± 0.26 ^c^	13.74 ± 1.55 ^b^	4.93 ± 0.90 ^c^
	Total free thiol groups (nmol/mg)	4.84 ± 1.65 ^a^	12.18 ± 2.46 ^b^	7.90 ± 1.41 ^c^	10.66 ± 1.22 ^d^	4.57 ± 2.23 ^a^
	Free amino nitrogen (µg/mg)	47.17 ± 2.32 ^ab^	51.35 ± 2.88 ^a^	48.62 ± 3.86 ^a^	45.48 ± 3.16 ^ab^	38.15 ± 1.14 ^b^

The proteins were extracted with option III without reduction and alkylation. For determination of exposed free thiol groups (A), the extracts were dissolved in 0.2 M Tris buffer; whereas to determine the total free thiol groups (B), 0.2 M Tris buffer containing SDS was used. Calibration was calculated using a series of concentrations of reduced glutathione and N-acetylcysteine. Data is expressed as means ± standard deviation (*n* = 3) in nmol/mg protein. SF: Santa Sofia Company; RC: Rio Colorado Company; DCB: de-pulped coffee beans; IFCB: initial fermentation coffee beans; FFCB: final fermentation coffee beans; WCB: washed coffee beans; GCB: green coffee beans. Different letters indicate significantly different values for each line (*p* < 0.05, ANOVA, Tukey’s test).

**Table 2:** Protein content after extraction options I, II, and III.

	mg Protein/100 mg DW		
	Option I	Option II	Option III
Santa Sofia	1.62 ± 0.04 ^a^	1.73 ± 0.19 ^a^	3.45 ± 0.13 ^b^
Rio Colorado	1.33 ± 0.21 ^a^	2.08 ± 0.08 ^b^	3.75 ± 0.04 ^c^

Option I: PVPP and ascorbic acid was used for the extraction; option II and III: SPS was used for the extraction. Data are expressed as means ± standard deviation (*n* = 3). Different letters indicate significantly different values within the lines (*p* < 0.05, ANOVA, Tukey’s).

## Data Availability

Not applicable.

## Supplementary Materials

The following are available online at https://www.mdpi.com/article/10.3390/foods11020159/s1, Figure S1. Schematic flowchart of the three protein extraction protocols used; Figure S2. Alignment of the data available for 11S from the UniProt database; Figure S3. Predicted structures of modifications by chlorogenic acid (CQA); Figure S4. Comparison of fluorescence emission spectra of protein extracts; Figure S5. Data of MALDI-TOF-MS measurements of the in-gel digested proteins; Table S1. Analyzed mass transitions of the P93079 sequence of the 11S protein.
