# Supplementary material for: Effect of the Post-Harvest Processing on Protein Modification in Green Coffee Beans by Phenolic Compounds
Source: Foods. 2022 Jan 8;11(2):159. doi: 10.3390/foods11020159 (PMC8775169; doi:10.3390/foods11020159)
Supplement: Supplementary file 1 [file foods-11-00159-s001.zip › foods-1524940-supplementary.pdf]

Supplementary Material

Effect of the post-harvest processing on the protein modification in green coffee beans by phenolic compounds

Gustavo A. Figueroa Campos [1], Johannes G. K. T. Kruizenga [1], Sorel Tchewonpi Sagu [1], Thomas Homann [1], Andreas Taubert [2], Harshadrai M. Rawel [1,*]

[1] Institute of Nutritional Science, University of Potsdam, Arthur-Scheunert-Allee 114-116, 14558 Nuthetal Germany; figueroacamp@uni-potsdam.de (G.A.F.C.), johanneskruizenga@web.de (J.G.K.T.K.), sorelsagu@uni-potsdam.de (S.T.S), homann@uni-potsdam.de (T.H.)

[2] Institute of Chemistry, University of Potsdam, Karl-Liebknecht-Str. 24–25, 14476 Potsdam, Germany; ataubert@uni-potsdam.de (A.T.)

* Corresponding author.

Tel: +49-33200-88-5525/5578 (H.M.R.)

E-mail: rawel@uni-potsdam.de

Content

Figure S1. Schematic flowchart of the three protein extraction protocols used. RC are samples from Rio Colorado; SF are samples from Santa Sofia; DPM are samples from different processing method; TCEP the reducing agent tris(2-carboxyethyl)phosphine and IAA the alkylation agent iodoacetamide

Figure S2. Alignment of the data available for 11S-CA from the UniProt database

Figure S3. Predicted structures of modifications by chlorogenic acid (CQA)

Figure S4. Comparison of fluorescence emission spectra of protein extracts. Results are presented as mean ± standard deviation (n = 3) as area under the curve (AUC). 1:1260 dilutions of the extracts dissolved in urea extraction buffer were excited at 360 nm light wavelength using fluorospectrometer and emission was measured between 300 and 500 nm. Extraction option I used PVPP, while extraction option II and III each used SDS. For the photo, 0.5 mL was pipetted onto a weighing dish in each case. Different letters indicate significant difference (p<0.05, ANOVA, Tukey test).

Figure S5. Data of MALDI-TOF-MS measurements of the in-gel digested proteins (a) Sequence coverage of peptic and tryptic in-gel digestion; (b) sequence coverage after extraction with options I and II. Data is expressed as means ± standard deviation (n = 3). Partials describe the incompletely digestion peptide based on the number of unused interfaces that are contained in the fragment spectrum of the in-silico digestion.

Table S1. Analyzed mass transitions of the P93079 sequence of the 11S protein

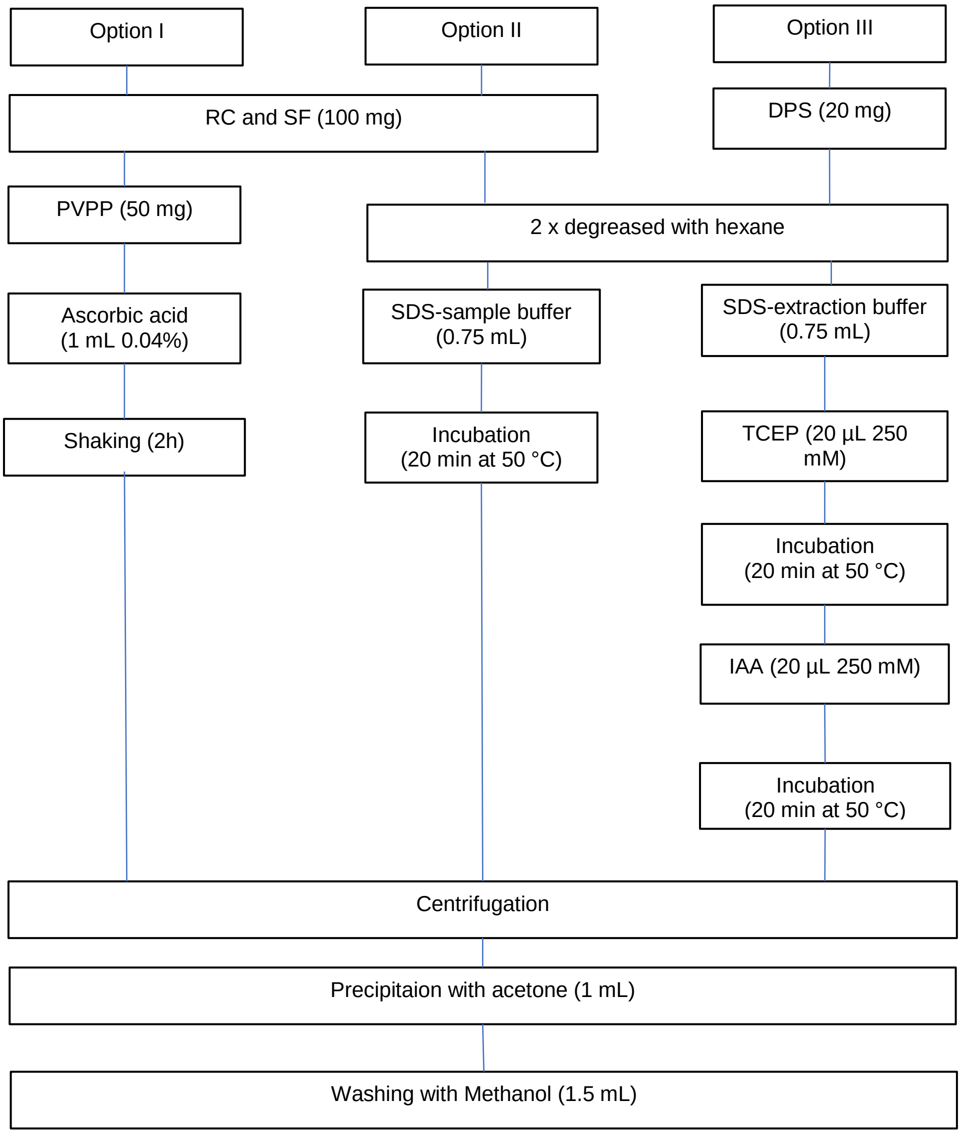

Figure S1. Schematic flowchart of the three protein extraction protocols used. RC are samples from Rio Colorado; SF are samples from Santa Sofia; DPM are samples from different processing method; TCEP the reducing agent tris(2-carboxyethyl)phosphine and IAA the alkylation agent iodoacetamide

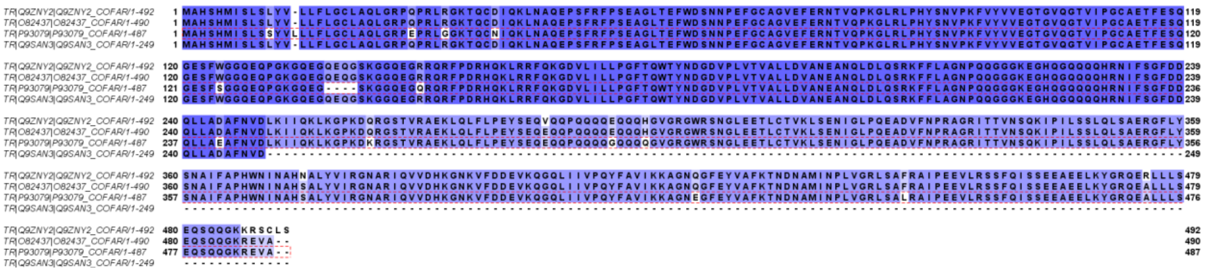

Figure S2. Alignment of the data available for 11S-CA from the UniProt database

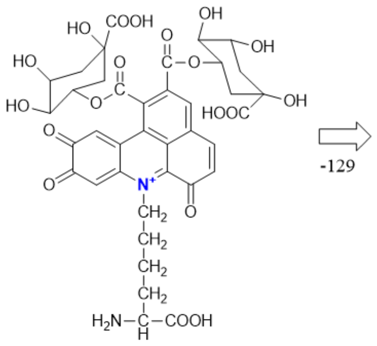

Mass gain 682 (-145 for Lys)
Dimer

CQA-1 (modified **Lys**)

Chemical Formula: $C_{38}H_{39}N_2O_{19}^{+}$
Exact Mass: 827.21

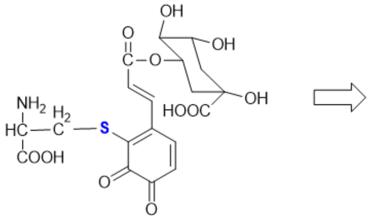

Mass gain 352 (-120 for Cys)
Monomer

CQA-2 (modified **Cys/Lys**)

Chemical Formula: $C_{19}H_{21}NO_{11}S$
Exact Mass: 471.08

Figure S3. Predicted structures of modifications by chlorogenic acid (CQA)

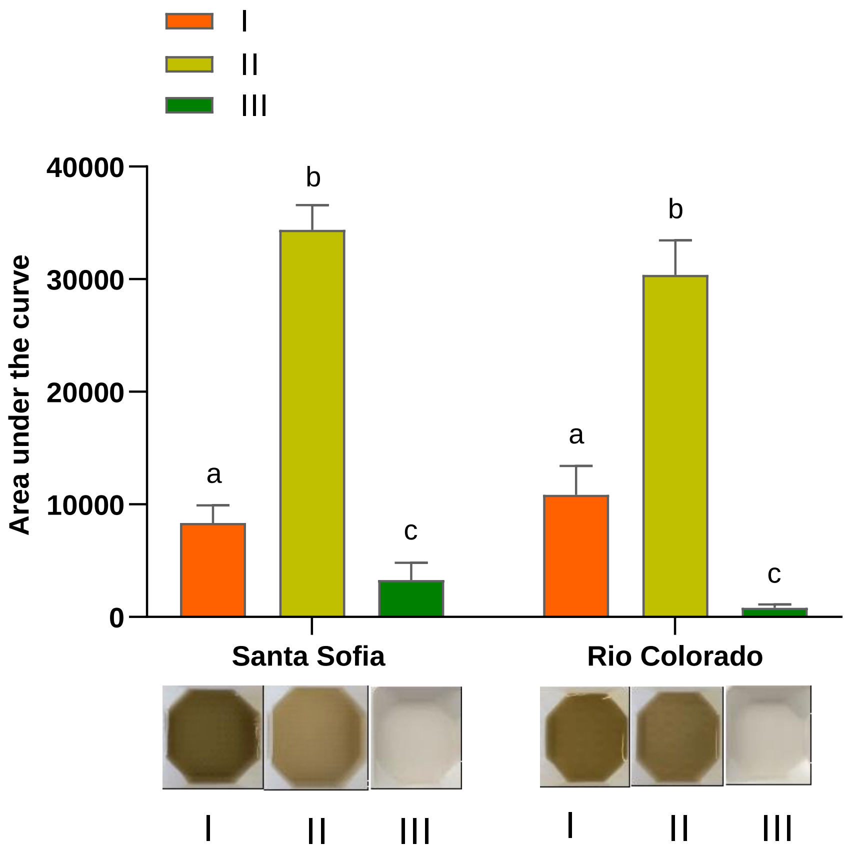

Figure S4. Comparison of fluorescence emission spectra of protein extracts. Results are presented as mean ± standard deviation (n = 3) as area under the curve (AUC). 1:1260 dilutions of the extracts dissolved in urea extraction buffer were excited at 360 nm light wavelength using fluorospectrometer and emission was measured between 300 and 500 nm. Extraction option I used PVPP, while extraction option II and III each used SDS. For the photo, 0.5 mL was pipetted onto a weighing dish in each case. Different letters indicate significant difference (*p*<0.05, ANOVA, Tukey test).

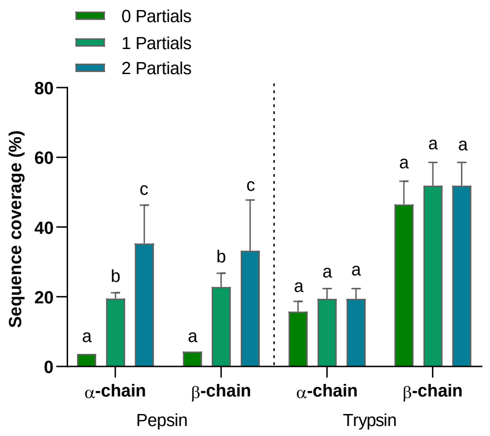

(a)

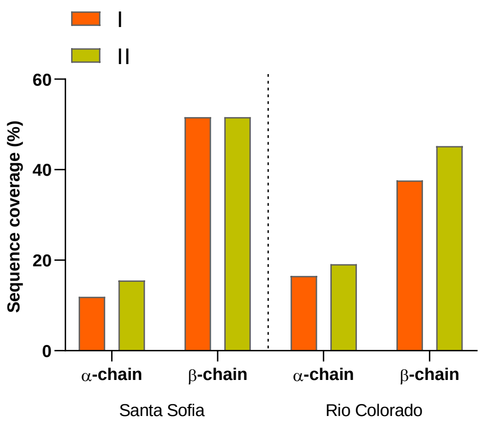

(b)

Figure S5. Data of MALDI-TOF-MS measurements of the in-gel digested proteins (a) Sequence coverage of peptic and tryptic in-gel digestion; (b) sequence coverage after extraction with options I and II. Data is expressed as means ± standard deviation (n = 3). Partials describe the incompletely digestion peptide based on the number of unused interfaces that are contained in the fragment spectrum of the in-silico digestion. Different letters indicate significantly different values for each group (different superscript letters indicate a significant different, $p < 0.05$, ANOVA, Tukey's test)

Table S1. Analyzed mass transition of the P93079 sequence of the 11S protein

	α-chain peptide sequence	Q1 (m/z)	Q3 (m/z)	Retention time (min)
Without modification of the lysine side chain	R.LGGK.T	187.6	261.1	4.2
			204.1	
			147.1	
	K.TQCNIQK.L	446.2	662.3	11.5
			502.3	
			388.3	
			275.2	
			147.1	
	K.LNAQEPSFR.F	531.3	834.4	14.2
			763.4	
			635.3	
			506.3	
			409.2	
			322.2	
	R.NTVQPK.G	343.7	572.3	11.1
			471.3	
			372.2	
	R.LPHYSNVPK.F	527.8	941.5	13.3
			707.4	
			544.3	
			457.3	
			343.2	
	K.GQEGSK.G	303.1	548.3	12.2
			420.2	
			291.2	
			234.1	
			147.1	
	R.FQK.G	211.6	275.2	8.8
			147.1	
	K.FFLAGMPQQGGK.E	660.8	785.4	15.4
			671.3	
			574.3	
			446.2	
			318.2	
	K.IIQK.L	251.2	388.3	11.2
			275.5	
			147.1	
CQA-Lysin adducts	R.LGGK.T	363.7	613.2	12.4
			556.2	
			499.2	
	R.LPHYSNVPK.F	703.8	1293.6	15.8
			896.4	
			809.4	
			695.3	
	K.GQEGSK.G	479.1	772.3	16.3
			643.2	
			586.2	
			499.2	
	K.FFLAGNPQQGGK.E	836.9	1137.5	20.4
			1023.4	
			926.4	
			798.3	
			670.3	
	K.IIQK.L	427.2	740.3	14.3
			627.3	
			499.2	
diCQA-Lysin adducts	R.LGGK.T	528.7	943.3	15.4
			886.3	
			829.2	
	K.TQCNIQK.L	787.3	1344.4	14.9
			1184.4	
			1070.4	
			957.3	
			829.2	

Table S1. Cont.

	β-chain peptide sequence	Q1 (m/z)	Q3 (m/z)	Retention time (min)
Without modification of the lysine side chain	K.LSEMIGLPQEADVFNPR.A	950.0	1172.6<br>632.4<br>386.2	16.8
	R.ITTVNSQK.I	445.8	676.4<br>575.3<br>476.2<br>362.2<br>529.3<br>616.3	11.9
	K.IPILSSLQLSAER.G	713.9	1313.7<br>903.5<br>816.5<br>703.4<br>575.3<br>462.2	17.4
	R.IQVVDHK.G	419.7	597.3<br>498.3<br>399.2<br>440.3<br>555.3	12.4
	K.VFDDEVK.Q	426.2	605.3<br>490.3<br>375.2<br>477.2<br>606.2	13.8
	K.AGNEGFEYVAFK.T	666.3	903.5<br>756.4<br>627.4<br>464.4<br>365.2	16.2
	K.TNDNAMINPLVGR.L	707.9	899.5<br>768.4<br>655.4<br>541.3	15.9
	R.LSALR.A	280.2	446.3<br>359.2<br>175.1	13.3
	R.AIPEEVLR.S	463.8	742.4<br>645.4<br>516.3	15.1
	R.SSFQISSEEAEELK.Y	792.4	934.4<br>847.4<br>718.3<br>589.3<br>518.2<br>389.2	15.4
	R.QEALLLSEQSQQGK.R	779.9	891.4<br>804.4<br>675.3<br>547.3<br>460.3<br>442.2<br>668.4	15.0

Table S1. Cont.

	β-chain peptide sequence	Q1 (m/z)	Q3 (m/z)	Retention time (min)
CQA-Lysin adducts	K.VFDDEVK.Q	602.3	727.3<br>598.3<br>499.2<br>477.2<br>606.2	17.0
diCQA-Lysin adducts	R.ITTVNSQK.I	786.8	1044.3<br>957.3<br>829.3<br>529.3<br>626.3	14.9
